# The influence of psychosocial work environment, personal perceived health and job crafting on nurses’ well-being: a cross-sectional survey study

**DOI:** 10.1186/s12912-024-02041-5

**Published:** 2024-06-03

**Authors:** Xin Zhang, Chen Zhang, Jiayan Gou, Shih-Yu Lee

**Affiliations:** 1 School of Nursing, Chinese Academy of Medical Sciences & Peking Union Medical College, Beijing, China; 2 Department of Nursing, Chinese Academy of Medical Sciences & Fuwai Hospital, Beijing, China; 3 School of Nursing, Hungkuang University, No. 1018, Sec. 6, Taiwan Boulevard, Taichung, 43302 Taiwan China

**Keywords:** Job crafting, Nurses, Psychosocial work environment, Well-being

## Abstract

**Background:**

The World Health Organization urged governments to prioritize the health and work well-being of nursing staff by promoting a positive working environment. A safe and healthy physical and psychosocial work environment is a basic human right for nurses. Job crafting is a necessary skill when facing challenging working conditions.

**Objectives:**

This cross-sectional correlational research based on the Job Demands-Resources Model aimed to explore the correlation between psychosocial work environment and work well-being among nurses working in the intensive care unit (ICU) and determine whether personal perceived health could mediate the relationship and whether job crafting can moderate the mediating effect. The study hypothesized that: 1. The psychosocial work environment would impact nurses' work well-being; 2. Personal perceived health would play a role as a mediator in the relationship between psychosocial work environment and work well‐being; 3. Job crafting would moderate the relationship between personal perceived health and work well‐being.

**Methods:**

A total of 655 registered nurses (RNs) from 7 ICUs in a teaching hospital in Beijing participated in this study. The RNs completed a battery questionnaire measuring their health, psychosocial work environment, well-being, and job crafting. PROCESS macros analysis was used to test mediating and moderating effects.

**Results:**

Personal perceived health mediated the relationship between psychosocial work environment and work well-being (b = 0.012, 95% CI [0.008, 0.016]). The moderated mediated analysis revealed that job crafting moderated perceived health’s impact on work well-being (b = -0.007, 95% CI [− 0.010, − 0.003]).

**Conclusion:**

A better psychosocial work environment with well-designed work organization and job content through job crafting could positively impact nurses’ health and work well-being.

## Introduction

“A safe and healthy working environment is a fundamental right at work,” said Gilbert F. Houngbo, the International Labour Organization (ILO) Director-General [1]. The World Health Organization (WHO) has also urged governments to recognize the importance of creating a positive working environment and committing to promoting the health and work well-being of nursing practitioners [2]. A positive working environment encompasses reducing absenteeism and improving retention rates. It is essential to understand that motivation plays a significant role in determining the quality of care [3]. An individual's work well-being is their perception of cognitive, emotional, health, and social conditions related to their profession [4]. A demanding work environment can quickly increase work-related stress, reduce work well-being, negatively impact physical and mental health, and lead to burnout [5–7]. About the psychosocial work environment factors (job demands, job control, and social support), previous researches have highlighted that workplace social support (coworker and supervisor support) is positively related to well-being but negatively related to burnout (high job demand and low job control) on health among healthcare workers [8, 9], In existing studies on nurses' well-being, the high-stress environment of the Intensive Care Unit (ICU) has been overlooked. However, study showed that ICU nurses often work extended hours and care for critically ill patients, which can result in physical and mental exhaustion, as well as burnout [10].

## Background

Over the past three years, most of the research on ICU nurses' struggles to adapt to challenging situations has been descriptive and primarily focused on external resources, revealing weaknesses in the healthcare systems in terms of staffing shortages, limited resources, lack of support, and access to training associated with pandemic [10–13]. Nursing staff in high-demand and low-resource work environments are particularly vulnerable to adverse impacts [14, 15]. These effects can lead to nursing staff withdrawing from the workplace, which in turn results in poor quality of care and impaired clinical decision-making [11, 13, 16] and ultimately jeopardizes patient safety [10]. Recently, studies suggest that implementating organizational citizenship behaviour among nurses can inhibit negative occurrence that may impact patients' health and well-being [17]. Furthermore, nurse differentiation and job crafting are effective retention strategies [18]. Overall, ICU nurses worldwide are not adequately prepared to handle the job demands brought on by the crisis, significantly impacting their ability to provide optimal patient care [19].

Based on the job demands‐resources theory (JD‐R) [20], which defines working conditions (job characteristics) from the perspective of job demands and job resources and includes two processes: the health-impairment process and the work-related incentive process-related well-being (consisting of burnout and work engagement). For individuals, a positive work environment is achieved through balancing job demands and resources [21]. Previous studies indicate that ICU nurses successfully utilize internal psychological mechanisms to adapt to work demands, switch job resources to cope with the situation, and maintain work well-being [6, 22, 23]. Some nurses utilized problem-focused coping strategies, such as seeking information and social support; others used emotion-focused coping methods, such as positive thinking, relaxation techniques, and peer support, to cope with challenging situations [24–26]. It is important to consider individual needs and situational factors when examining nurses' ability to adapt to work demands, as the work environment can positively and negatively impact nurses.

Job crafting refers to an active process in which employees redefine and shape their roles to increase motivation and engagement in their tasks [27]. Job crafting involves employees proactively adjusting the demands and resources of their jobs to align with their preferences and strengths. Individuals and the work environment can influence job crafting as individuals modify their behaviors to balance work demands and resources [28]. Ensuring a healthy environment in the workplace can enhance productivity, safety, and comfort for employees [29]. Research confirmed that job crafting training is an effective method to assist employees in aligning job demands and resources. This can increase work engagement and performance, reduce fatigue, and improve health outcomes [30]. There has been an increase in ward-level crafting which is related to an individual-level increase in work engagement. Nursing managers who create an environment that encourages crafting task boundaries for team members’ growth are likely to contribute to increasing nurses' work engagement [31]. For ICU nurses, job crafting can be a valuable skill to help cope with workload and balance work and personal life in the workplace. According to recent studies, individuals who experience positive emotions, exhibit higher job satisfaction, and lower levels of burnout are more likely to thrive in their workplace [32, 33]. Job crafting is a crucial factor that protects nurses from the adverse effects of work stress and burnout. It can significantly improve their well-being and create a positive psychosocial work environment. However, our understanding of job-crafting behaviors among ICU nurses is limited, and we still need to determine the individual and environmental factors that influence job crafting.

This study aimed to explore how personal perceived health (self-rated health, burnout, stress), as well as the psychosocial work environment (demands at work, work organization and job contents, interpersonal relations and leadership, and work-individual interface), relate to work well-being and job crafting among ICU nurses. Based on the JD‐R model [5, 34], the secondary purpose was to explore the potential mechanism of this connection with personal perceived health as a mediator and job crafting as a moderator. We hypothesized that: 1. the psychosocial work environment would impact nurses' work well-being. 2. Nurses' personal perceived health would play a role as a mediator in the relationship between psychosocial work environment and work well‐being. 3. Job crafting would moderate the relationship between personal perceived health and work well‐being.

## Method

### Study design and participants

This cross-sectional correlational exploratory study recruited participants from 7 ICUs of a medical center in Beijing with 12-h rotating shifts. The inclusion criteria were Registered Nurses (RN) providing direct patient care in the ICU and nurses who self-reported being pregnant with a history of mental illness were excluded. Based on the N:q rule [35] in the context of Structural Equation Modeling, it is appropriate to have N:q values ranging from 10:1 (10 observations per one estimated parameter) to even 20:1 seem appropriate. In this study, there are 12 estimated parameters (N:q values = 20:1), which means that 240 participants were needed. From May to August 2022, a convenient sample of 655 ICU RNs was recruited, and the study was completed. The Institutional Review Board approved the study at the research site (No. 2022030).

### Measures

#### Sociodemographic

Sociodemographic data included age, gender, educational level, marital status, work hours, and length of time as a nurse and in the current ICU.

#### Job crafting

The Job Crafting Scale (JCS) [36] was used to assess job crafting, with a 21-item, 5-point Likert-type scale (1 = never, 5 = very often), where a higher mean score indicates higher job crafting behaviors. The JCS comprises four subscales that measure four aspects of job crafting, including increasing structural work resources (*n* = 5), decreasing hindering job demands (*n* = 6), increasing social job resources (*n *= 5), and increasing challenging job demands (*n* = 5). The Cronbach's alpha of the JCS was 0.89 [36]. The Chinese version of the JCS was unavailable, so the first author (XZ) obtained permission from Tims and put together a team to translate the JCS. They followed the recommended procedures for cross-cultural research [37] and used forward- and backward-translate [38] methods to ensure that the original and target language conveyed the same meaning and content equivalence. The Cronbach's alpha of the Chinese JCS was 0.89 in the present study, and the four subscales ranged from 0.85 to 0.91.

#### Psychosocial work environment and personal perceived health

The Copenhagen Psychosocial Questionnaire (COPSOQ) II-short Chinese version [39, 40] was utilized in this study as a 40-item Likert-type scale to evaluate the psychosocial work environment and personal health. The psychosocial work environment was assessed in Part 1 through four dimensions: (1) demands at work, (2) work organization and job contents, (3) interpersonal relations and leadership, and (4) work-individual interface. Personal perceived health was assessed in Part 2 through three dimensions: (1) self-rated health, (2) burnout, and (3) stress. The intensity or frequency of each question was measured on a score range of 0 to 100, where high scores in part 1 subscales indicate favorable psychosocial factors. In contrast, high scores in part 2 subscales suggest unfavorable factors. For example, a higher self-rated health indicates poor health conditions. The Cronbach's alpha of the COPSOQ was 0.83 in the current study [39, 40].

#### Work well-being

The work well-being was measured by the Workplace Well-Being (WWB), a subscale of the Employ Well-being scale [41]. The WWB comprises six items scored on a 7-point Likert-type scale (1 = totally disagree, 7 = totally agree), with higher scores indicating higher levels of work well-being. The Cronbach's alpha of this subscale was 0.93 in the current study [41].

### Data collection

The Nursing department at the research site used WeChat, a Chinese social media app, to send recruitment messages to ICU nurses. All the potential participants were informed that whether they participated in the study, they would not lose any benefits as an employee at the hospital, and completing the questionnaires was entirely voluntary. Nurses won't receive any benefit from their participation. Informed consent forms and completed questionnaires were submitted through a secure online platform (www.wjx.cn). All the contact information and questionnaire data were stored separately to ensure anonymity and confidentiality.

### Data analysis

Data were analyzed using the SPSS 24.0 version. All data were evaluated to ensure statistical assumptions were met before substantive analysis. Before data analysis, all data were evaluated to ensure that all met statistical assumptions. Descriptive statistics were used to describe sample characteristics and the main variables, including mean ± standard deviation (SD), median (P_25_, P_75_), range, and percentage. Pearson's correlation analyses explored the associations between the dependent variable (work well-being) and the independent variables. We utilized Hayes' PROCESS macro program [42] and bootstrapping to assess the significance of the moderated mediation model, which is a reliable method for evaluating the magnitude of conditional indirect effects [43]. According to our hypothesized model, we used Model 14 to examine the indirect effects of the psychosocial work environment on work well‐being through personal perceived health and job crafting. We used the bias-corrected bootstrapping method to generate 5000 random samples with a 95% bias corrected confidence interval (CI). Bootstrap CIs were used to determine the statistical significance of Model 14. If the CIs did not contain zero, the effect was considered significant.

## Results

### Descriptive of the study participants and the variables

The mean age of the 655 study participants (Table 1) was 30.22 years old (*SD* = 5.48). The majority of them were female (92.5%), single (41.8%), and with a bachelor's or higher degree (72.4%). They had 7 (median) years of RN experience and 6 (median) years of current ICU experience. All study participants worked 12-h shifts (day and night within a week). Theoretically, education could impact an individual’s perceptions and behaviors. Therefore, the population was divided into two groups: diploma and at least college education. We compared the differences between the two groups to examine the potential impact of education on individuals' job crafting and perceived health (self-rated health, burnout, and stress). However, there is no significant difference (*p* > 0.05). Their perceptions of the psychosocial work environment (M = 65.05, *SD* = 8.45) and personal perceived health (M = 45.31, *SD* = 20.89) were moderate; however, their work well-being and job crafting scores were at a higher level (Table 2). Each variable’s skewness ranged from − 1.35 to 0.35, while kurtosis ranged from − 1.01 to 1.77. According to the reference values of an absolute skewness value ≤ 2 or an absolute kurtosis ≤ 4, the normality assumptions of the variables were met.

**Table 1:** Characteristics of the study participants (*N* = 655)

Variable	Min ~ Max	Mean ± SD	N (%)
**Age (years)**	21 ~ 52	30.22 ± 5.48	
Years in hospital*	1 ~ 32	7.0 (4, 11)	
Years in ICU*	0.5 ~ 27	6.0 (2, 10)	
**Gender**			
Male			49 (7.5)
Female			606 (92.5)
**Education**			
Diploma			181 (27.6)
College & MSN			474 (72.4)
**Marriage**			
Married			381 (58.2)
Single			274 (41.8)

Note. All the median (P_25_, P_75_)

**Table 2:** Characteristics of psychosocial work environment, personal perceived health, work well-being, and job crafting (*N* = 655)

Variable	Min ~ Max	Mean ± SD
**Psychosocial work environment**	33.02 ~ 90.36	65.05 ± 8.45
COP subscale 1-demands at work	8.33 ~ 91.67	46.09 ± 13.26
COP subscale 2-work organizations and job contents	25 ~ 100	70.24 ± 12.51
COP subscale 3-interpersonal relations and leadership	15 ~ 100	74.32 ± 16.09
COP subscale 4-work-individual interface	19.78 ~ 100	69.57 ± 10.16
**Personal perceived health**	0 ~ 100	45.31 ± 20.89
Self-rated poor health	0 ~ 100	42.29 ± 28.60
Burnout	0 ~ 100	46.64 ± 22.22
Stress	0 ~ 100	47.00 ± 21.40
**Job crafting**	1.81 ~ 5	3.69 ± 0.61
Increasing structural job resources	1 ~ 5	4.21 ± 0.69
Increasing social job resources	1 ~ 5	3.56 ± 0.79
Increasing job demands	1 ~ 5	3.24 ± 0.88
Decreasing job demands	1 ~ 5	3.73 ± 0.68
**Work well-being**	1.67 ~ 7	6.17 ± 0.95

Note. All the median (P_25_, P_75_)

### Linking personal perceived health, job crafting, and psychosocial work environment to work well-being: The moderated mediation model

Socidemographics were not significantly associated with the dependent and independent variables. The correlations between independent variables (personal perceived health, psychosocial work environment, job crafting), and dependent variable (work well-being) are detailed in Table 3.

**Table 3:** Correlations among psychosocial work environment, personal perceived health, work well-being, and job crafting (*N* = 655)

Variables	Work well-being	1	2	3	4	5	6	7
1. Job crafting	0.417**	—	—	—	—	—	—	—
2. COP subscale 1	-0.334**	-0.201**	—	—	—	—	—	—
3. COP subscale 2	0.453**	0.400**	-0.145**	—	—	—	—	—
4. COP subscale 3	0.528**	0.453**	-0.209**	0.689**	—	—	—	—
5. COP subscale 4	0.345**	0.279**	-0.087*	0.478**	0.641**	—	—	—
6. Self-rated poor health	-0.384**	-0.256**	0.363**	-0.325**	-0.348**	-0.190**	—	—
7. Burnout	-0.413**	-0.242**	0.483**	-0.289**	-0.319**	-0.071	0.582**	—
8. Stress	-0.394**	-0.208**	0.489**	-0.245**	-0.292**	-0.067	0.527**	0.797**

Note. * *p* < 0.05, ** *p* < 0.01. COP subscale 1 = demands at work; COP subscale 2 = work organizations and job contents; COP subscale 3 = interpersonal relations and leadership; COP subscale 4 = work-individual interface

**Fig. 1:**
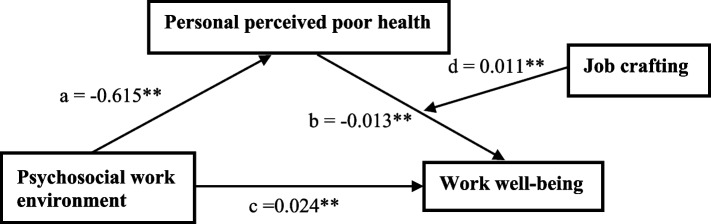
The final moderated mediation model. Note. ** *p *< 0.01. Psychosocial work environment: including COP subscale 2 = work organizations and job contents; COP subscale 3 = interpersonal relations and leadership. Path a = the effect of psychosocial work environment dependency on personal perceived poor health, path b = the effect of personal perceived poor health dependency on work well-being, path c = the effect of psychosocial work environment dependency on work well‐being, path d = the conditional effect of job crafting on personal perceived poor health to work well‐being

We aimed to determine whether the link between psychosocial work environment (COP subscale 1–4) and work well-being was mediated by personal perceived health (M) and whether job crafting (W) support influenced this relationship (Table 4). After controlling for the effect of mediator and moderator, it was found that a positive and significant relationship existed between the psychosocial work environment (including COP subscale 2 = work organizations and job contents and COP subscale 3 = interpersonal relations and leadership) and work well-being (*b* = 0.024, *p* < 0.001). The findings support Hypothesis 1. The analysis showed that the poor psychosocial work environment was significantly associated with personal perceived health (*b* = -0.615, *p* < 0.001). Furthermore, better personal perceived health (M) was linked to high levels of work well-being (*b* = 0.021, *p* < 0.001). There was a positive indirect effect of the psychosocial work environment on work well-being through better personal perceived health (M) (*b* = 0.012, *95% CI* [0.008, 0.016]). The findings support Hypothesis 2. The indexes of moderated mediation were found to be significant (*b* = -0.007, *95% CI* [− 0.010, − 0.003]). This suggests that the effect of personal perceived health on work well-being varies with job crafting (W). In the moderated mediation model, the interaction between personal perceived health and job crafting was significant (*b* = 0.011, *95% CI* [0.006, 0.015]). The findings support Hypothesis 3. The final moderated mediation model (R^2^ = 0.41, F = 113.77, *p* < 0.01) is presented in Fig. 1.

**Table 4:** Direct and indirect effects of the moderated mediation models (PROCESS)

**Variables**			**Effect/**	**SE**	***p***	**BC Bootstrap 95% CI**	
			**Index**			Lower	Upper
Direct effect	Psychosocial work environment → Work well-being		0.024	0.003	0.000	0.019	0.029
	Psychosocial work environment → Personal perceived poor health		-0.615	0.057	0.000	-0.728	-0.503
	Personal perceived poor health → Work well-being		-0.013	0.002	0.000	-0.016	-0.010
	Job crafting → Work well-being		0.276	0.053	0.000	0.172	0.380
	Personal poor perceived health * Job crafting		0.011	0.002	0.000	0.006	0.015
Indirect effect	Psychosocial work environment → Personal perceived poor health → Work well-being						
			0.012	0.002		0.008	0.016
Index of moderated mediation			-0.007	0.002		-0.010	-0.003

Note. *SE* standard error. Psychosocial work environment: including COP subscale 2 = work organizations and job contents**;** COP subscale 3 = interpersonal relations and leadership

## Discussion

The study was conducted after the 3rd year of the COVID‐19 pandemic, capturing the effects of the context on ICU nurses' work well‐being. The findings revealed that a positive psychosocial work environment was associated with nurses' work well‐being (Hypothesis 1), and personal perceived health might mediate this association (Hypothesis 2). Moreover, job crafting could moderate the association between personal perceived health and work well‐being for ICU nurses (Hypothesis 3). This study's work well-being and job crafting level among ICU nurses is slightly higher than other studies [44–46]. The majority of the study participants were female, which is in line with the current nursing workforce in China. It has been identified that gender can influence thinking style [47]; therefore, conducting gender-specific studies could help prevent gender bias and lead to the development of tailored-made interventions to enhance work well-being. This finding suggests that the nurses in this study can redesign their work to better align with their skills and needs, enabling them to adapt and respond to changes and challenges in their work environment more effectively [48].

### Encourage job crafting by recognizing personal successes and patient progress

The study’s findings have demonstrated that the psychosocial work environment significantly impacts the health and well-being of ICU nurses. The higher scores of increasing structural job resources and reduced job demands mean that ICU nurses can actively strive for work autonomy and have high expectations and requirements for reducing unnecessary duplication of work and excessive non-nursing work. This highlights the importance of nursing managers optimizing workflow to improve work efficiency and provide nurses with adequate resources [49]. Besides, the lowest score for increased job demands is consistent with findings from multiple studies [45, 50, 51], which may be related to a heavy ICU workload, critically ill patients, nurses tending to be stable and safe, and a lack of willingness to take the initiative to face challenges. ICU nurses may feel tired and stressed after encountering high job demands, requiring more interest and motivation for more challenging tasks.

The previous study showed that a structured psychosocial work environment with well-designed work organization and job content (WOJC) can improve psychological health and well-being, productivity, and retention [52]. The study showed that a WOJC that aligns with the workers' capabilities and skills can benefit both individuals and the organization [52]. According to the current study, the "work organizations and job contents" significantly positively impact work well-being (Fig. 1). Furthermore, most nurses believe that their professional identity comes from recognizing patients and their disease outcomes, which drives the redesigned behaviors of ICU nurses' work. Nursing managers should encourage their staff to achieve individual success and provide training on improving work efficiency by managing their time effectively and optimizing workflow. It is important for nurses to constantly learn new skills, knowledge, and techniques to cope with potential stress and challenges that may arise in their work. The findings of this study also suggest that "interpersonal relations and leadership" in the work environment positively affect ICU nurses' work well-being. Although work well-being is self-perceived by ICU nurses, its occurrence and mechanism of action are mainly influenced by the leadership style of nursing managers [46]. If the leadership style is more inclusive, open, and practical, it can improve job performance by increasing personal work engagement and work well-being.

### Job crafting can decrease the adverse impact of the work environment on personal health

The results of this study suggest that ICU nurses’ personal perceived health can have a mediating effect on their psychological work environment and work well-being. That means nurses' work well-being can be indirectly improved by improving the work environment through enhancing personal resources. ICU nurses face numerous challenges, such as fast-paced nursing work, high pressure, frequent night shifts, prolonged standing, and managing critically ill patients with variable medical conditions. These challenges result in both physical and psychological stress [53]. Given the adverse impact of the work environment on nurses' health, we suggest setting up a proactive system that can promptly identify, report, and monitor health-related outcomes. To achieve this, a thorough and rigorous evaluation, efficient referral mechanisms, and conservative treatment options must be established to facilitate a safe return to work. Besides, it is imperative to have sufficient staffing and well-equipped facilities to ensure the health and safety of nurses in the workplace. Finally, according to a WHO global report, clear communication and leadership are also among the social determinants of health [54].

The results also show that job crafting moderates the relationship between personal perceived health and work well-being. Job crafting can moderate the mediated pathway between psychosocial work environment, personal perceived health, and work well-being. It can be understood that job crafting allows ICU nurses to make adaptive adjustments when facing different work environments. If the psychosocial work environment influences an individual's perceived health, job crafting can be considered an adaptive strategy to help individuals better acclimate to such environments. Due to positive individual factors, ICU nurses who engage in job crafting behaviors experience better-perceived health and increased ability to cope with stress and burnout. In the JD-R model, nurses can use their resources to acquire more work resources by actively participating in training and communicating with external parties [55]. Research showed that nurses with high levels of self-efficacy are confident, proactive, and utilize their strengths to conduct job crafting and seek resources at work [56–58]. Previous studies [59, 60] have shown that intervention programs such as job-crafting diaries can effectively enhance ICU nurses' self-efficacy and improve their job-crafting behaviors. In addition, it is important to encourage ICU nurses to participate in the decision-making process. Enhancing their voice and sense of participation is essential for improving their work engagement.

### Effective supervisor support create a conducive workplace

Research confirmed that transformational and servant leadership styles have a significant positive correlation with job crafting, while authoritarian leadership has a significant negative correlation [61, 62]. Research also showed young nurses tend to score higher in job crafting, which suggests that they are more dedicated to their work [63, 64]. This is particularly true for new nurses, who are often more curious and enthusiastic about their work environment and its content. They are likelier to maintain a positive attitude, actively seek new learning opportunities, and explore the workplace. It is recommended that nursing managers pay attention to individual nurse characteristics, encourage job crafting among young nurses, and provide personalized training and support to improve the alignment between individuals and the job roles. Senior ICU nurses have extensive clinical experience and are highly adaptable. Managers should provide more career development opportunities to stimulate their work enthusiasm and motivation. Nursing managers should adopt an effective leadership style and provide nurses with the necessary resources and conditions that create a conducive work environment. They should also demonstrate tolerance, support, and guidance toward nurses' job-crafting behaviors. By doing so, they can maximize the potential effectiveness of individual job crafting and achieve a win–win situation for both individuals and organizations. Ultimately, this will help to improve the quality of ICU nursing care and medical services.

### Implications for practice

Based on our research findings, ICU nurses can adopt positive job crafting behaviors, such as flexibly adjusting work styles, organizing work tasks, and actively adapting to changes in the work environment to better maintain individual health perception and improve work well-being. In addition, we recommend that nursing managers prioritize and enhance the psychosocial work environment, transforming leadership styles to be more transformative and service-oriented. And we advocate for the encouragement and support of nurses in job crafting to make changes in their job, elevating their autonomy and adaptability through training and communication initiatives and make their jobs more meaningful. We propose establishing a health monitoring and support system coupled with timely health intervention measures to enhance individual perceived health. These suggestions are poised to assist in optimizing nurses' work experiences and improving their overall well-being in practice.

### Limitations

Although this study provides valuable insights into the work well-being of ICU nurses, it’s important to note that the results are based on a single data collection time point, which may limit their internal validity and generalizability. Secondly, selection bias is a common limitation in cross-sectional studies that could affect the causality of observed relationships. Therefore, future longitudinal studies are needed to confirm the findings. Thirdly, there may be bias introduced by excluding nurses who are pregnant or have a history of mental illness. To enhance the robustness of the findings, it is recommended that future studies be conducted with multiple sites and longitudinal research.

## Conclusion

The study found that nurses’ work well-being was positive influenced by the good psychosocial work environment, and negitive influenced by their poor health. Job crafting behaviors could also modulate the pathway from nurses' perceived health to work well-being. ICU nurses who engaged in job crafting behaviors were more likely to experience positive work well-being, which could help them proactively manage work stress and challenges. Further research is needed to identify effective strategies for promoting job-crafting behaviors among ICU nurses.

## Data Availability

The datasets generated and/or analysed during the present study are not publicly available due to the data being proprietary and confidential records of Chinese Academy of Medical Sciences & Peking Union Medical College, but are available from the corresponding author on reasonable request.
